# mRNA-LNP vaccination-based immunotherapy augments CD8^+^ T cell responses against HPV-positive oropharyngeal cancer

**DOI:** 10.1038/s41541-023-00733-8

**Published:** 2023-09-29

**Authors:** Ke Qiu, Xing Duan, Minzi Mao, Yao Song, Yufang Rao, Danni Cheng, Lan Feng, Xiuli Shao, Chuanhuan Jiang, Hai Huang, Yan Wang, Huifang Li, Xuemei Chen, Sisi Wu, Dan Luo, Fei Chen, Xingchen Peng, Yongbo Zheng, Haiyang Wang, Jun Liu, Yu Zhao, Xiangrong Song, Jianjun Ren

**Affiliations:** 1 Department of Otolaryngology-Head & Neck Surgery and Department of Critical Care Medicine, Frontiers Science Center for Disease-related Molecular Network, State Key Laboratory of Biotherapy and Cancer Center, West China Hospital, Sichuan University, Chengdu, Sichuan China; 2 Research Core Facility of West China Hospital, Sichuan University, Chengdu, Sichuan China; 3 Department of Biotherapy and National Clinical Research Center for Geriatrics, Cancer Center, West China Hospital, Sichuan University, Chengdu, Sichuan China

**Keywords:** RNA vaccines, Cancer immunotherapy

## Abstract

Although mRNA vaccines are known as potent activators of antigen-specific immune responses against infectious diseases, limited understanding of how they drive the functional commitment of CD8^+^ T cells in tumor microenvironment (TME) and secondary lymphoid organs hinders their broader application in cancer immunotherapy. Here, we systematically evaluated the immunological effects of a lipid nanoparticle (LNP)-encapsulated mRNA vaccine that encodes human papillomavirus E7 protein (HPV mRNA-LNP), a tumor-specific antigen of HPV-positive oropharyngeal squamous cell carcinoma (OPSCC). HPV mRNA-LNP vaccination activated overall and HPV-specific CD8^+^ T cells, as well as differentially drove the functional commitment of CD8^+^ T cells through distinct IFN-response and exhaustion trajectories in the spleen and TME, respectively. Combination therapies of HPV mRNA-LNP vaccination with immune checkpoint blockades boosted HPV-specific CD8^+^ T cells while maintaining their anti-tumor function, thus further promoting tumor regression. Our results showed that the HPV mRNA-LNP vaccination combined with immune checkpoint blockade is a promising approach for immunotherapy of HPV-positive OPSCC.

## Introduction

Antigen-specific CD8^+^ T cells are the major components of the tumor-reactive immune system, and their quality and magnitude largely determine the anti-tumor efficiency of immunotherapy^1,2^. Several types of CD8^+^ T cell-targeting immunotherapies, including immune checkpoint inhibitors (ICIs), chimeric antigen receptor-modified T cells (CAR-T), and T-cell receptor-gene-engineered T cells (TCR-T), show remarkable efficacy in treating certain neoplasms. However, their application is significantly limited by the unavoidable systemic adverse effects, heterogeneous treatment effects, and high cost^3–7^.

The emergence of mRNA vaccines has enabled significant advancements in vaccine technology, which could be tailored to translate any protein antigen without the risk of host genomic integration^8^. However, naked mRNA can be degraded rapidly once it enters the body, and delivery vectors are required for effective uptake by target cells^9^. Recent developments in lipid nanoparticle (LNP) delivery systems offer a promising platform for in vivo mRNA delivery, overcoming the issues of instability and toxicity^10^. LNP-encapsulated mRNA (mRNA-LNP) vaccines were found to hold incredible therapeutic potential in several types of solid tumors. However, multiple steps are involved in vaccination-induced anti-tumor immunity^11,12^, and how they drive the functional commitment of CD8^+^ T cells in the tumor microenvironment (TME) and secondary lymphoid organs remains elusive.

It’s also noteworthy that, to properly simulate clinical situations, therapeutic tumor vaccines are usually applied when the tumor has reached a reasonable size^13^. During this process, CD8^+^ T cells are exposed to persistent antigen stimulation, and they gradually acquire exhaustion-related features; which not only represented a highly activated and functional states, but also provide backdoors for immunosuppressive TME to trigger the lack of persistence of effector CD8^+^ T cells^14,15^. Therefore, further identification of the key inhibitory molecules that can be targeted to enhance the mRNA-LNP vaccination-induced CD8^+^ T-cell immune response might further inform the rational design of vaccine formulations and contribute to the development of effective combination therapies.

In this study, we constructed a mRNA-LNP vaccine encoding human papillomavirus (HPV)-16 E7 protein (HPV mRNA-LNP), and aimed to (1) determine the optimal route of vaccination for inducing an efficacious overall and HPV-specific CD8^+^ T cell immune responses; (2) evaluate its anti-tumor effects in HPV-positive oropharyngeal squamous cell carcinoma (OPSCC); (3) systematically demonstrate its influence on the immune landscape and functional commitment of CD8^+^ T cells; (4) investigate whether its combination with immune checkpoint blockades could further enhance the anti-tumor efficiency.

## Results

### Formulation and characterization of HPV mRNA-LNPs

**Fig. 1:**
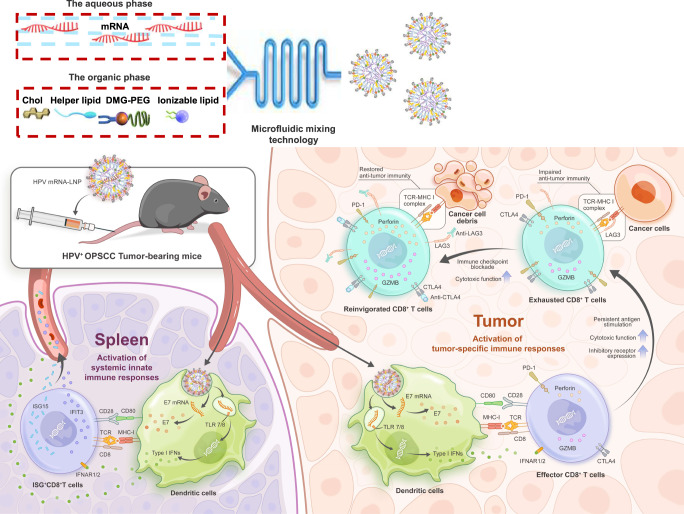
Schematic representation of the formation and immunological mechanism of HPV mRNA-LNPs. Active tumor targeting was achieved through both the activation of systemic innate immune responses and tumor-specific immune responses. Once entering the body, on one hand, HPV mRNA-LNPs were uptaken by dendritic cells in the spleen and stimulate a significant release of type I IFNs for the activation of systemic innate immune responses. On the other hand, HPV mRNA-LNPs were uptaken by dendritic cells in the tumor microenvironment and activate tumor-specific effector immune responses. Nevertheless, persistent antigen stimulation resulted in CD8^+^ T cell exhaustion, which is characterized by gradually increasing expression of multiple inhibitory receptor genes, providing a backdoor for the functional impairment of effector cells. Therefore, combination therapy of HPV mRNA-LNP vaccination with immune checkpoint blockade boosted effector CD8^+^ T cells while restoring their anti-tumor function, thus further promoting tumor regression.

**Fig. 2:**
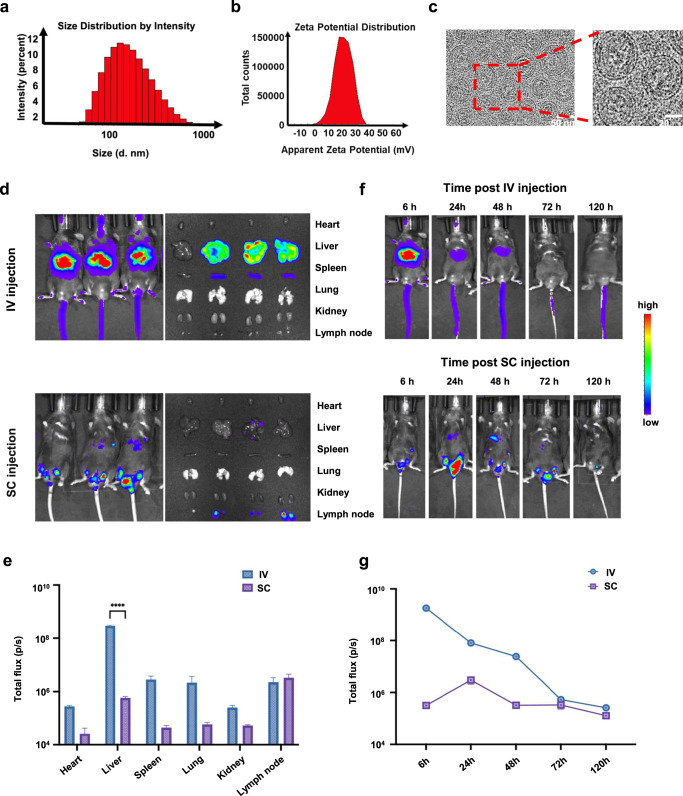
Characterization of mRNA-LNPs. **a** Size number and **b** Zeta potential distribution of LNPs. **c** Cryo-TEM images of LNPs. **d** Biodistribution and **e** Quantitative analysis of luciferase expression in mice after 6 h following IV and SC administration of LNP@Luc-mRNA vaccine (*n* = 3). **f** Biodistribution and **g** Quantitative analysis of luciferase expression in mice over time following IV (left) and SC (right) vaccination with LNP@Luc-mRNA (Error bar = mean ± SEM, *n* = 3). Statistics were assessed by one-way ANOVA with Tukey’s multiple comparison tests. *P < 0.05 was considered statistically significant. **P < 0.01 and ***P < 0.001 were considered highly significant. LNP lipid nanoparticle, IV intravenous, SC subcutaneous, LNP@Luc-mRNA nanoparticles loaded with mRNA encoding luciferase.

The preparation and proposed structure of the HPV mRNA-LNPs are shown in Fig. 1. Dynamic light scattering (DLS) demonstrated the formation of HPV mRNA-LNPs with a mean hydrodynamic diameter of 117.67 nm (Fig. 2a). The mean zeta potential of HPV mRNA-LNPs was 17.67 mV (Fig. 2b), which was consistent with the presence of cationic lipids. Cryo-transmission electron microscopy (cryo-TEM) confirmed that HPV mRNA-LNPs had a relatively homogeneous spherical shape with an electron-dense internal amorphous structure and an exterior multilayer (Fig. 2c).

We further used luciferase mRNA (mLuc) as a model mRNA for tracking the in vivo distribution of the expressed protein. Six hours later, bioluminescence was used to detect the location of protein expression (Fig. 2d, e). Following intravenous (IV) injection, fluorescent proteins were detected in the liver, spleen, lungs, and lymph nodes. However, following subcutaneous (SC) injection, the expression of fluorescent proteins was mainly confined to the lymph nodes. We then monitored the protein expression over time (Fig. 2f, g). It was noteworthy that, following IV injection, the expression reached the maximum after 6 h and declined slowly afterward, whereas following SC injection, the protein expression peaked at 24 h and dropped rapidly.

Collectively, these findings indicate that mRNA can be well encapsulated in LNPs and constantly translated into target proteins over a relatively long period.

### Optimization of vaccination routes for inducing efficacious overall and HPV-specific CD8^+^ T cell immune responses

**Fig. 3:**
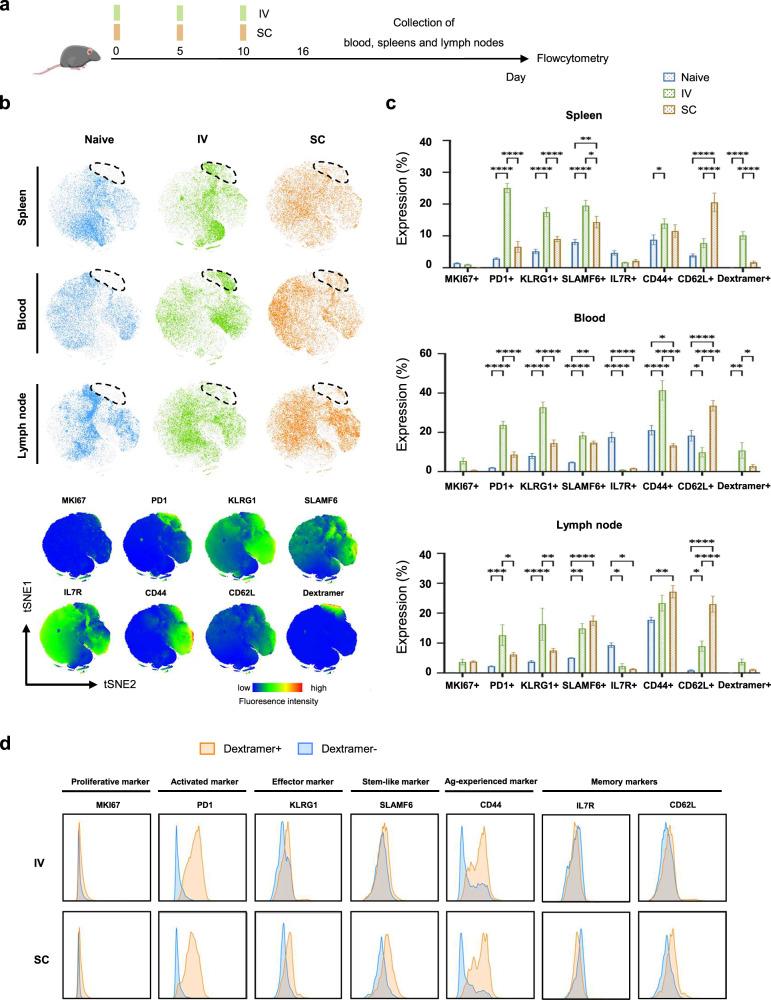
Optimization of vaccination routes for inducing efficacious overall and HPV-specific CD8^+^ T cell immune responses. **a** C57BL/6J mice (*n* = 5) were vaccinated subcutaneously or intravenously (10 μg/100 μl) on days 0, 5, and 10 with mRNA-LNPs. Whole blood, spleen, and inguinal lymph nodes (LNs) were collected on day 16 to measure the frequency of overall and dextramer^+^ CD8^+^ T cells. **b** T-SNE maps showing cluster distribution of CD8^+^ T cells using flowcytometry data (top) and t-SNE heatmaps for each marker applied on CD8^+^ T cell events (bottom). **c** Expression of different immune markers on CD8^+^ T cells in spleen, blood, and inguinal LNs in (Error bar = mean ± SEM, *n* = 5). Statistics were assessed by two-way ANOVA with Tukey’s multiple comparison tests. *P < 0.05, **P < 0.01 and ***P < 0.001. **d** Histograms showing the differential expression of phenotypic markers expressed by dextramer^+^ or dextramer- populations after LNP-IV (top) or LNP-SC (bottom) assessed using flow cytometry (concatenated, *n* = 15). Data are representative of three independent experiments. Naive unvaccinated, IV intravenous, SC subcutaneous.

To determine the optimal route of vaccination for inducing efficacious overall and HPV-specific CD8^+^ T-cell immune responses, C57BL/6J non-tumor bearing (TB) mice were subcutaneously and intravenously vaccinated with HPV mRNA-LNPs. The spleens, whole blood, and lymph nodes were collected 6 days after the third vaccination (Fig. 3a). We systematically characterized the phenotypes of CD8^+^ T cells induced by IV and SC vaccination. IV vaccination-induced CD8^+^ T cells were committed to a more activated (PD1^+^) and effector-like (KLRG1^+^) phenotype, whereas SC vaccination-induced CD8^+^ T cells were more likely to display a memory-like phenotype (CD62L^+^), regardless of tissue origin (Fig. 3b, c). HPV-specific CD8^+^ T cell responses were characterized using the H-2D_b_- RAHYNVTF peptide– dextramer. A more significant expansion of HPV-specific CD8^+^ T cells was observed in the spleen and blood following IV vaccination compared to that following SC vaccination (Fig. 3b, c and Supplementary Fig. 1a). Meanwhile, HPV-specific CD8^+^ T cells induced by both IV and SC vaccinations were more activated, effector-like, and antigen-experienced than their nonspecific counterparts (Fig. 3d).

Collectively, these findings suggest that IV vaccination generates a more robust expansion of HPV-specific CD8^+^ T cells, which are functionally superior to their non-specific counterparts.

### ScRNA-seq of CD8^+^ T cells demonstrates IFN-induced and exhausted cell accumulation induced by HPV mRNA-LNP vaccination

**Fig. 4:**
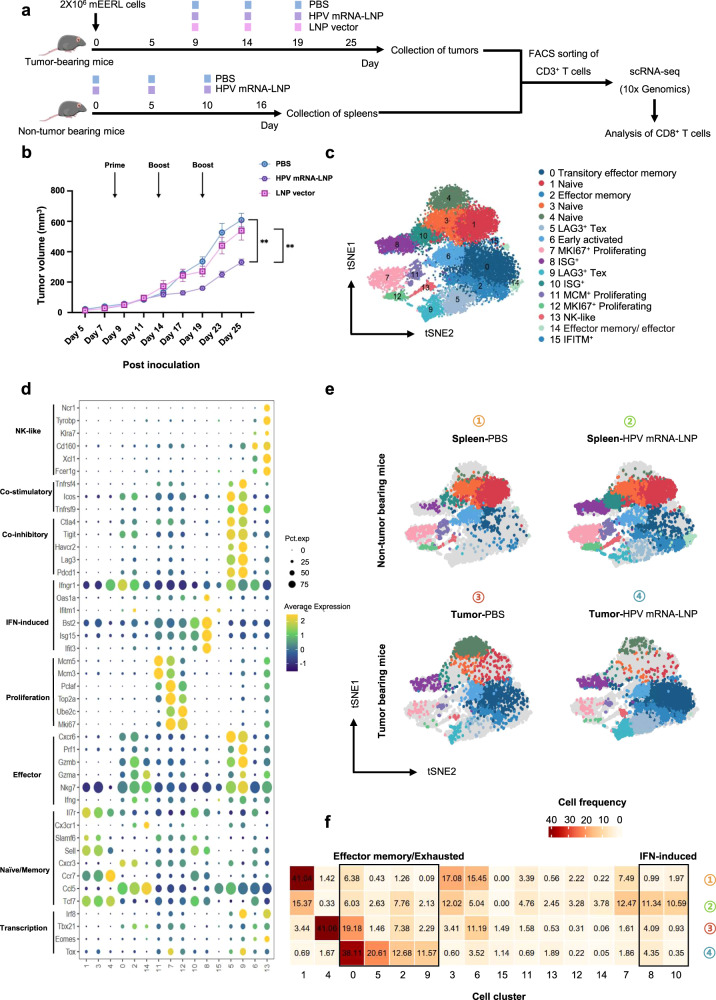
ScRNA-seq of CD8^+^ T cells demonstrates IFN-induced and exhausted cell accumulation induced by HPV mRNA-LNP vaccination. **a** Schematic of the therapeutic study design. Mice were implanted with mEERL cell line and treated with HPV mRNA-LNPs on day 9, 14, and 19 (*n* = 10). CD3^+^ T cells in tumors from TB mice were sorted using flow cytometry on day 25 (6 days after the third dose of vaccination). CD3^+^ T cells in spleens from non-TB mice were also sorted using flow cytometry on day 16 (6 days after the third dose of vaccination) as control. **b** Tumor growth following treatment (Error bar = mean ± SEM, *n* = 10). Statistics were assessed by one-way ANOVA with Tukey’s multiple comparison tests. *P < 0.05, **P < 0.01 and ***P < 0.001. **c** Distribution of sorted CD8^+^ T cells projected onto t-SNE map. **d** Dot plot of canonical markers identifying specific CD8^+^ T cell subsets. **e** T-SNE maps of CD8^+^ T cell subsets in the spleens of non-TB mice and the tumors of TB mice, respectively. **f** Heatmap of cluster-wise cell frequencies for each combination of treatment and tissue. Cell frequency was presented as the percentage of each cluster in all cells (the sum of the values in each row is 100%). TB tumor bearing.

To investigate the anti-tumor efficiency of HPV mRNA-LNP vaccination, mice were challenged with HPV^+^ OPSCC and vaccinated intravenously (Fig. 4a). Tumor growth was significantly inhibited in mice vaccinated with HPV mRNA-LNPs, compared to that in mice mock-vaccinated with PBS and mice vaccinated with empty LNPs (Fig. 4b).

To characterize the vaccination-induced alterations in CD8^+^ T cells, CD3^+^ T cells in tumors from TB mice mock-vaccinated with PBS and their counterparts vaccinated with HPV mRNA-LNPs were sorted using flow cytometry on day 25 (6 days after the third dose of vaccination). CD3^+^ T cells in the spleens of non-TB mice mock-vaccinated with PBS and their counterparts vaccinated with HPV mRNA-LNPs were also sorted using flow cytometry on day 16 (6 days after the third dose of vaccination) as a control. The sorted CD3^+^ T cells were subjected to scRNA-seq, in which CD8^+^ T cells were extracted for subsequent analyses. A total of 16 cell clusters consisting of 19,938 CD8^+^ T cells were identified based on their unique gene signatures, including naïve (clusters 1, 3 and 4), early activated (cluster 6), effector memory (clusters 0, 2, and 14), IFN-stimulated gene (ISG)^+^ (clusters 8, 10, and 15), proliferating (clusters 7, 11, and 12), NK-like (cluster 13), and exhausted (clusters 5 and 9) clusters (Fig. 4c, d, Supplementary Fig. 2a and Supplementary Data 1). Naïve cells (clusters 1, 3 and 4) were characterized by the expression of canonical naïve markers *Tcf7* (encoding TCF1), *Ccr7* (encoding CCR7) and *Sell* (encoding CD62L). Cluster 6 was characterized by the low expression of naïve marker genes (*Tcf7* and *Sell*) and moderate expression of both *Ifngr1* (encoding interferon gamma receptor 1) and *Ccl5* (encoding C-C motif chemokine ligand 5), suggesting a state of early activation^16^. Clusters 7, 11, and 12 exhibited high proliferative potential. Cluster 11 specifically expressed genes required for DNA replication (*Mcm3* and *Mcm5*), whereas clusters 7 and 12 specifically expressed genes related to mitotic processes (*Mki67*, *Ube2c*, and *Top2a*), indicating that they may belong to different phases of the cell cycle. Cluster 13 was characterized by high expression of NK-like genes (*Ncr1*, *Klra7*, *Cd160*, and *Fcer1g*) and moderate expression of effector-like genes (*Cxcr6*, *Prf1*, *Gzma*, and *Gzmb*); therefore, it was defined as an NK-like subcluster. Clusters 0, 2, and 14 expressed both *Ccl5* and canonical effector-like genes (*Cxcr6*, *Prf1*, *Gzma*, and *Gzmb*), constituting a group of effector memory cells with varying effector functions. In addition to highly specified effector gene signatures, clusters 5 and 9 displayed high expression levels of inhibitory receptor genes (*Pdcd1*, *Lag3*, *Ctla4*, *Tigit*, and *Havcr2*), consistent with the exhausted subpopulation (Tex)^17–19^. In addition, we also unexpectedly identified three T cell subclusters with high expression of ISGs, which have been demonstrated to be responsible for quick immune responses in infectious diseases but are rarely reported in tumor settings^20,21^. These sub-clusters were further recategorized into five modules based on the expression patterns of functional genes, including proliferation, exhaustion, IFN-response, effector/memory, and naive (Supplementary Fig. 2b).

To further assess whether cells from specific clusters were derived from different tissues or whether they were induced by HPV mRNA-LNP vaccination, we examined the cluster-wise cell distribution across tissues and treatments (Fig. 4e–f). CD8^+^ T cells derived from the spleens of non-TB mice mock-vaccinated with PBS were mainly localized in naïve clusters (58%), whereas CD8^+^ T cells derived from the spleens of non-TB mice vaccinated with HPV mRNA-LNPs had a lower frequency in naïve clusters (27%); they were mainly localized in the IFN-induced clusters (22%), indicating the rapid initiation of innate immune responses^22,23^. CD8^+^ T cells derived from tumors of TB mice mock-vaccinated with PBS were predominantly found in the naïve cluster (41%), and to a lesser extent, in the effector memory clusters (27%). Nevertheless, in TB mice vaccinated with HPV mRNA-LNPs, tumor-infiltrated CD8^+^ T cells had an obviously lower frequency in the naïve cluster (1.67%) and were mainly localized in the effector memory clusters (51%) and exhausted clusters (32%), suggesting initiation of the exhaustion process under continuous exposure to antigens^18^.

Collectively, scRNA-seq of CD8^+^ T cells demonstrated that HPV mRNA-LNP vaccination could augment innate immune responses in the spleen, while also contributing to effector specification and exhaustion of CD8^+^ T cells in the TME.

### HPV mRNA-LNP vaccination drives the functional commitment of CD8^+^ T cells through two distinct trajectories: IFN-response and exhaustion

**Fig. 5:**
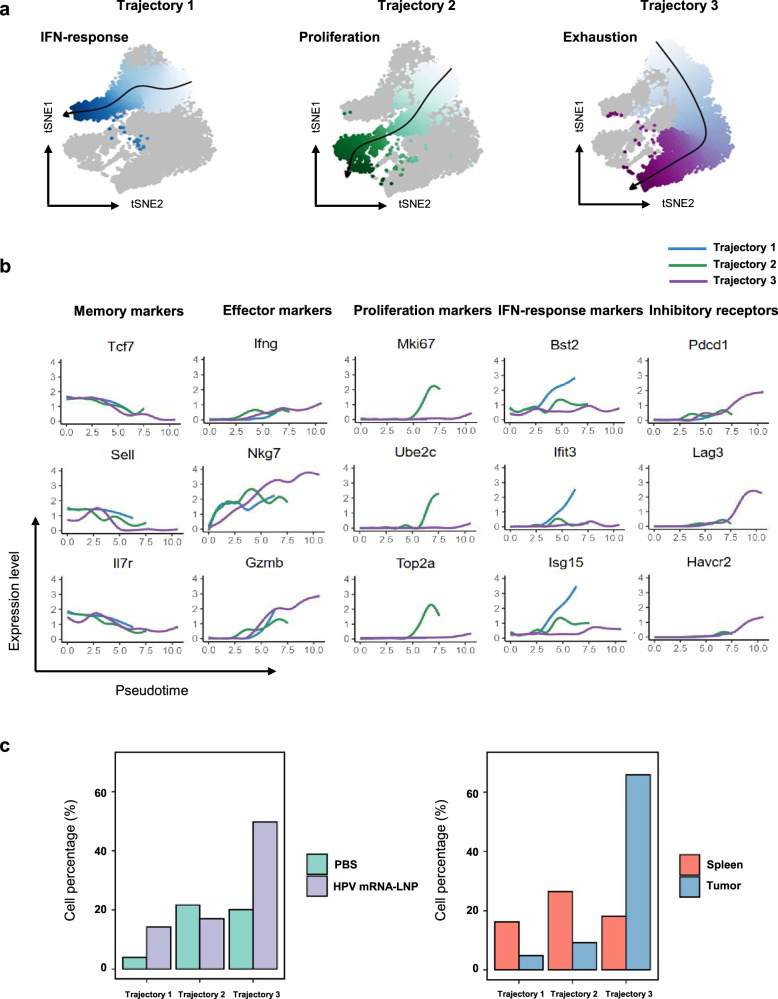
HPV mRNA-LNP vaccination drives the functional commitment of CD8^+^ T cells through IFN-response and exhaustion trajectories. **a** Differentiation trajectories inferred using pseudotime and projected onto t-SNE maps. **b** Expression of inhibitory receptors, memory, effector, proliferation, and IFN-response markers along with three pseudotime trajectories. **c** Bar plots of cell percentages across treatments (left) and tissues (right) for each trajectory. Data is derived from the therapeutic study in Fig. 4.

scRNA-seq transcriptomic profiling demonstrated different immune phenotypes between the spleens and tumors, suggesting distinct stages of cell differentiation. To further investigate whether CD8^+^ T cells undergo distinct differentiation trajectories following HPV mRNA-LNP vaccination, we traced the transcriptional alterations corresponding to their functional commitment. Three distinct differentiation trajectories were inferred using pseudo-time analysis (Fig. 5a). The first trajectory originated from the naïve cluster and progressed to ISG^+^ clusters, thus was related to the activation of IFN signaling pathway with increased expression of IFN-stimulated genes (Fig. 5a left and Fig. 5b). The second trajectory also originated from the naïve cluster but progressed through the early activated cluster and ended in the proliferating clusters, along which an increase in the expression of cell cycle-related genes was observed (Fig. 5a middle and Fig. 5b). The third trajectory originated from the naïve memory cluster, passed through the effector memory clusters, and progressed to the exhausted clusters. Accordingly, effector and inhibitory marker genes exhibited a progressive increase in expression from naïve cells towards the exhaustion stage (Fig. 5a right and Fig. 5b).

CD8^+^ T cells from mice mock-vaccinated with PBS were predominantly distributed in the proliferation and exhaustion trajectories, whereas an increase in cell proportions in both the IFN-response and exhaustion trajectories was observed in CD8^+^ T cells from mice vaccinated with HPV mRNA-LNPs (Fig. 5c, left and Supplementary Data 2). Meanwhile, CD8^+^ T cells from the spleen were distributed in roughly equal proportions among the three trajectories, while their counterparts from the tumors were predominantly enriched in the exhaustion trajectory (Fig. 5c, right and Supplementary Data 2).

Collectively, HPV mRNA-LNP vaccination drives the functional commitment of CD8^+^ T cells through two unique differentiation trajectories: IFN-response and exhaustion.

### HPV mRNA-LNP vaccination induces robust TCR clonotype expansion in effector and exhausted cell subclusters

**Fig. 6:**
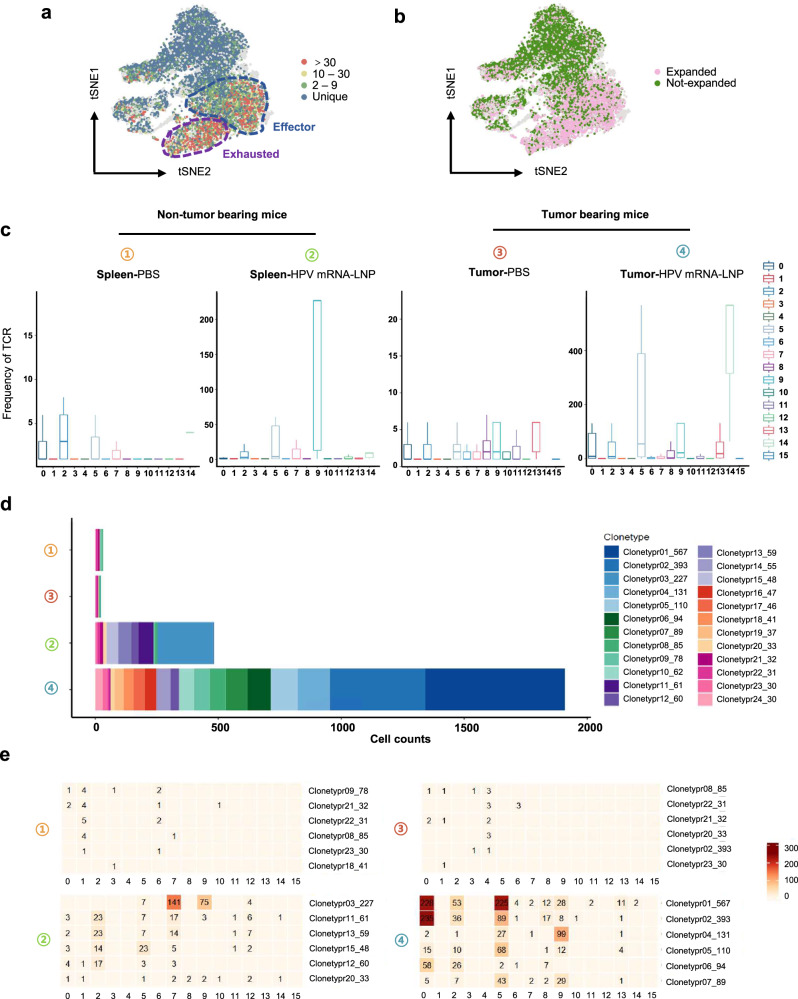
HPV mRNA-LNP vaccination induces robust TCR clonotype expansion in effector and exhausted cell subsets. **a** T cell receptor (TCR) clonotype size of CD8^+^ T cells projected onto t-SNE. Blue circle indicates effector subsets, purple circle indicates exhausted subsets. **b** Distribution of expanded (clone size ＞ 1) and non-expanded (clone size = 1) TCR clonotype of CD8^+^ T cells projected onto t-SNE. **c** Distribution of TCR cell frequencies among clusters measured in the spleens and tumors, respectively. The median is shown by the center line within the box of box plots, while the lower and upper box boundaries reflect the 25th and 75th percentiles, respectively, with whiskers drawn to the number that is closest to but still falls within the 1.5 interquartile range from top to bottom of the box borders. **d** Distribution of TCR clonotype (clone size ≥30) size of CD8^+^ T cells in the spleens and tumors. The TCR label indicates the numeric ID of each clonotype (the former number) and the number of cells belonging to the clonotype (the latter number), respectively. **e** TCR-wise cell counts of top clonotypes across clusters in the spleens and tumors, respectively. Spleens were harvested from non-TB mice and tumors were harvested from TB. Data is derived from the therapeutic study in Fig. 4. TB tumor bearing.

To further investigate whether the increased proportions of effector and exhausted CD8^+^ T cells were related to their clonal expansion, we integrated the scRNA-seq and scTCR-seq data to analyze TCR sequences at the single-cell level. Unique TCRs were mainly identified in the naïve and early activated subclusters, whereas hyper-expanded clonotypes (clone size >30 cells) were predominantly found in the effector memory and exhausted subclusters (Fig. 6a, b). An increase in the proportion of clonally expanded cells in the naïve, effector memory, and exhausted subclusters further validated the progressive differentiation in trajectory 3 (Fig. 6c and Supplementary Fig. 3a).

Moreover, HPV mRNA-LNP vaccination generated distinct TCR patterns in the spleen and tumor (Fig. 6d and Supplementary Data 3; TCRs with clone sizes >30 are shown). Cells in both the spleens of non-TB mice and tumors of TB mice mock-vaccinated with PBS displayed almost no hyperexpanded TCR sequences, whereas broader TCR clonotypes were observed in their counterparts vaccinated with HPV mRNA-LNPs, in which the highest number of hyperexpanded TCR clones was detected in the tumors. We assessed the cluster-wise distribution of TCR clones across tissues and treatments (Fig. 6e; the top six expanded clonotypes in each group are shown). The hyperexpanded clonotype 03 was predominant in the cells from the spleens of non-TB mice vaccinated with HPV mRNA-LNPs, which were mainly enriched in the proliferating and exhausted subclusters. Whereas hyperexpanded clonotypes 01, 02, and 04 were predominant in the cells from the tumors of TB mice vaccinated with HPV mRNA-LNPs, which were mainly enriched in the effector memory and exhausted subclusters (Fig. 6e, Supplementary Fig. 3b and Supplementary Data 4).

Collectively, vaccination-induced clonal expansion is mainly enriched in tumor-infiltrated effectors and exhausted cell subclusters, which could be the main reactive components in anti-tumor immunity.

### HPV mRNA-LNP vaccination combined with immune checkpoint blockade promotes tumor regression without increasing systemic adverse effects

**Fig. 7:**
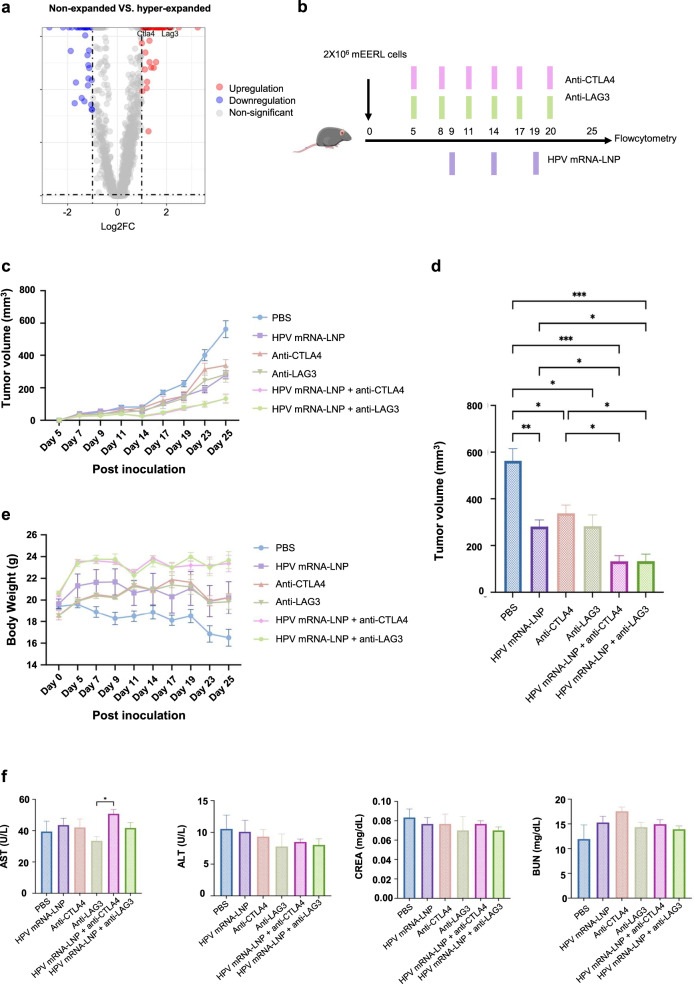
HPV mRNA-LNP vaccination combined with immune checkpoint blockade promotes tumor regression without increasing systemic adverse effects. **a** Volcano plot showing significantly (*p* value < 0.05) upregulated (log2fold change > 1, red) or downregulated (log2fold change < −1, blue) genes of clonally hyperexpanded (clone size ≥ 30) CD8^+^ T cells compared to that in non^-^expanded (clone size = 1) CD8^+^ T cells. **b** Schematic of therapeutic study design. Mice were implanted with mEERL cell line and vaccinated intravenously (10 μg/100 μl) on day 9, 14, and 19. Immune checkpoint inhibitors (ICIs) were administered intraperitoneally every 3 days (200 μg per treatment for anti-LAG3; 100 μg per treatment for anti-CTLA4). Whole blood, spleen, tumor, and draining lymph nodes (DLNs) were collected on day 25 for subsequent experiments. **c**, **d** Tumor growth following treatment (*n* = 5–8). Statistics were assessed by one-way ANOVA with Tukey’s multiple comparison tests. *P < 0.05, **P < 0.01 and ***P < 0.001. **e** Body weight change following treatment (*n* = 5–8). **f** Serum levels of alanine aminotransferase (ALT), aspartate aminotransferase (AST), creatinine (CREA), and blood urea nitrogen (BUN) following treatment (*n* = 5–6). Statistics were assessed using Tukey’s multiple comparison tests. Error bar = mean ± SEM.

Considering that exhausted cell subclusters may be the main tumor-reactive components induced by HPV mRNA-LNP vaccination, we hypothesized that upregulated inhibitory receptor genes not only represented a highly activated and functional states, but also provide backdoors for immunosuppressive TME to trigger the lack of persistence of effector CD8^+^ T cells. We initially applied the most commonly used PD-1 blockade in HPV^+^ OPSCC mouse model. However, the results showed that PD-1 blockade-induced tumor regression was not statistically significant (Supplementary Fig. 4). Therefore, we then systematically compared the gene expression profiles of clonally hyperexpanded (clone size >30) and non-expanded CD8^+^ T cells (clone size = 1) and identified *Lag3* and *Ctla4* as the most significantly upregulated inhibitory receptor genes (Fig. 7a). To investigate the anti-tumor efficiency of HPV mRNA-LNP vaccination combined with immune checkpoint blockade (ICB), TB mice were vaccinated intravenously with or without ICB (Fig. 7b). As expected, in contrast to HPV mRNA-LNP vaccination alone or ICB alone, the combination of HPV mRNA-LNP vaccination and LAG3/CTLA4 blockade exacerbated synergistic effects on tumor growth (Fig. 7c, d, Supplementary Fig. 5a). To further assess the systemic adverse effects of these combination therapies, we monitored the weight change of mice in each treatment group. And we found that TB mice mock-vaccinated with PBS experienced a gradual decline in body weight, whereas the body weight of TB mice receiving the combination therapies remained relatively stable, suggesting of good tolerability (Fig. 7e). Besides, serum levels of alanine aminotransferase (ALT), blood urea nitrogen (BUN), aspartate aminotransferase (AST), and creatinine (CREA) were measured on day 25. Notably, no significant kidney or liver injury was detected in mice receiving the combination therapy (Fig. 7f).

**Fig. 8:**
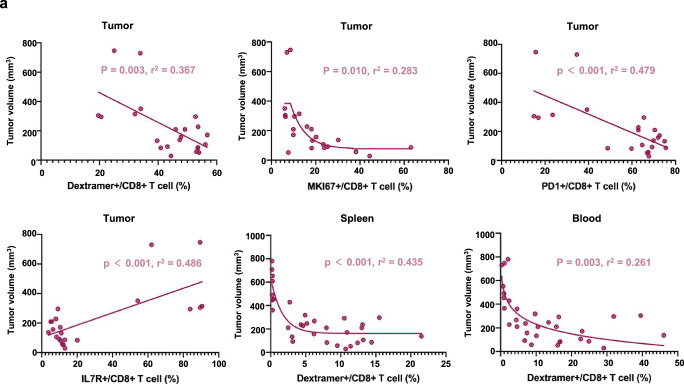
The abundance of HPV-specific CD8^+^ T cells correlates with superior antitumor capacity. **a** Correlation between tumor volume and immune cell frequencies on day 25. Correlations were assessed using Pearson correlation coefficients, *P* < 0.05 and R^2^ > 0.25 were considered significant.

We systematically characterized the phenotypes of CD8^+^ T cells induced by combination therapies. In contrast to HPV mRNA-LNP vaccination alone, combination therapy with LAG3 significantly increased the proportion of HPV-specific CD8^+^ T cells in the blood as well as stimulated a more proliferative (MKI67^+^) phenotype in the TME (Supplementary Fig. 5b, c and Supplementary Fig. 6a, b). In addition, the proportion of HPV-specific CD8^+^ T cells in the spleen, blood, and TME, but not in draining lymph nodes (DLN), was negatively correlated with tumor volume (Fig. 8a).

Overall, these findings suggest that HPV mRNA-LNP vaccination combined with immune checkpoint blockade promotes tumor regression without increasing systemic adverse effects.

## Discussion

Antigen-specific CD8^+^ T cells are major components of the tumor-reactive immune system, and their quality and magnitude largely determine the anti-tumor efficiency of immunotherapy^1^. Therapeutic mRNA vaccines are effective in inducing robust antigen-specific CD8^+^ T cell expansion in several preclinical models^24^, whereas how they drive the functional commitment of CD8^+^ T cells in the TME and secondary lymphoid organs remains elusive. Here, we provide solid evidence in mouse models that systemic vaccination with HPV mRNA-LNPs could induce robust expansion of both overall and HPV-specific CD8^+^ T cells, directing them towards IFN-response and exhaustion differentiation trajectories, thereby improving their anti-tumor efficiency.

mRNA vaccines encapsulated by LNP or Lipoplexes have already been developed in preclinical models and in humans, with varied mRNA delivery systems based on whether self-developed or existing ionizable lipids^25–27^. These studies have provided some essential clues for the core delivery vector of mRNA, prescription optimization and clinical application. However, none of these delivery systems have been approved for clinical practice. Given that our work aims to answer some key questions regarding the previously unknown process of the functional commitment of CD8^+^ T cells induced by mRNA-LNP vaccination, and provide molecular and cellular basis for the improvement of its anti-tumor efficiency; therefore, we chose the most representative mRNA vaccine delivery system based on DLin-MC3-DMA, which is the first approved RNA delivery vector by FDA^28^, and we believe it can provide more convincing data and is of considerable referential importance for mRNA vaccine mechanistic research.

Initially, we showed that the route of administration could modify the magnitude and quality of both the overall and HPV-specific CD8^+^ T cells. IV vaccination generated more activated and effector-like cells than SC vaccination, resulting in more memory-like cells. In addition, IV vaccination induced a more robust expansion of HPV-specific CD8^+^ T cells. These findings can be partially explained by previous reports that systemic immunity is required in the therapeutic setting of cancer immunotherapy^29^. Specifically, the spleen is considered the primary site for the interaction of dendritic cells (DCs) with T cells following systemic vaccination, in which tumor antigens rapidly stimulate a significant release of type I IFNs, prepare DCs for cross-presentation, and ultimately activate antigen-specific T cells^30,31^. Consistently, our scRNA-seq analysis identified three unique ISG^+^ CD8^+^ T cell subclusters that displayed high expression of IFN-stimulated genes and were enriched in the spleen following IV vaccination, suggesting that potent systemic innate immune activation was induced by HPV mRNA-LNP. Therefore, the systemic vaccination with HPV mRNA-LNP could not only generate antigen-specific CD8^+^ T cells, but also activate type I IFN signaling pathway required for T cell activation.

It is noteworthy that exhausted T cells identified in our work are not truly dysfunctional cells, but rather a group of activated and functional effector cells. A previous study based on different syngeneic tumor models has shown that CD8^+^ T cells co-expressing inhibitory receptors (IRs) are not dysfunctional, but rather highly express activation and effector-related marker genes such as IFNG, GZMB, MKI67, and ICOS. Besides, their abundance was positively associated with tumor control and response to PD-L1 blockade^32^. Meanwhile, in a study focusing on melanoma, CD8^+^ T cells co-expressing IRs were found to be highly clonally expanded and proliferative^33^. Similarly, in this study, we found that cells undergoing exhaustion trajectory were characterized by a progressive increase in the expression of both effector and inhibitory marker genes, and acted as the main reactive components in anti-tumor immunity. Collectively, these findings strongly indicate that Tex cells are a heterogeneous population of not only dysfunctional but also highly activated and functional effector cells.

Nevertheless, our findings dose not contradict the fact that the engagement of these IRs with their ligands can provide backdoors for immunosuppressive TME to trigger the lack of persistence of effector CD8^+^ T cells. Rather, as expected, ICB can maintain the functionality of these Tex cells by blocking the corresponding inhibitory signaling pathways. The generation of antigen-specific CD8^+^ T cells is the primary goal of many tumor vaccines; however, it is not sufficient for achieving satisfactory therapeutic efficacy. In several phase I/II studies, the application of therapeutic tumor vaccines alone showed frustrating outcomes in combating recurrent or metastatic HPV-associated cancers^34,35^, indicating that monotherapy with therapeutic tumor vaccines to treat HPV-associated cancers requires improvement. In addition, increasing evidence in the past decade has shown that persistent antigen stimulation can result in CD8^+^ T cell exhaustion, which is characterized by gradually increasing expression of multiple inhibitory receptor genes, providing a backdoor for the functional impairment of effector cells via multiple immunosuppressive mechanisms^18^. Therefore, further identification of the key inhibitory molecules that can be targeted to enhance the mRNA-LNP vaccination-induced CD8^+^ T-cell immune response might further contribute to the development of effective combination therapies. In this study, CD8^+^ T cells in the TME differentiated through an exhaustion trajectory following HPV mRNA-LNP vaccination. We then systematically compared the gene expression profiles of clonally hyperexpanded and non-expanded CD8^+^ T cells and identified *Lag3* and *Ctla4* as the most significantly upregulated inhibitory receptor genes. As expected, subsequent combination therapy with HPV mRNA-LNP vaccination and immune checkpoint blockade (anti-LAG3/anti-CTLA4) further promoted tumor regression. Meanwhile, mice receiving the combination therapy did not suffer from significant kidney or liver injury, indicating that it might be safe and tolerable.

The benefits of pairing these two mechanistically different immunotherapies have also been demonstrated in other studies. In a preclinical study, adenovirus vector-based vaccine was found to result in an overall benefit in HPV^+^ TB mice, and its subsequent combination with PD-1 antibody further promoted tumor regression^36^. In addition, similar synergistic anti-tumor effects against HPV-associated cancers have also been revealed in animal studies focusing on the combination of live-attenuated bacterial vector vaccines or DC-based vaccines with ICB, indicating a rationale for pairing therapeutic tumor vaccines and ICB^37,38^. Meanwhile, recent clinical trials have further confirmed the benefits from this combination. In a phase II trial with ISA101 (a HPV-16 peptide vaccine) and nivolumab (a PD-1 blocking antibody) for patients with advanced HPV-16^+^ tumors, a 2-year overall survival (OS) rate of 33% and a median OS of 15.3 months were reported^39^. Besides, a phase Ib/IIa trial with similar design combining MEDI0457 (an HPV vaccine plus IL-12) with durvalumab (a PD-L1 blocking antibody) in patients with recurrent/metastatic HPV^+^ HNSCC reported an objective response rate (ORR) of 27.6% and a median OS of 29.2 months^40^. These results are encouraging when compared with a phase III trial using nivolumab monotherapy, which showed a median OS of 9.1 months and 2-year OS rate of 16.9% among patients with HPV^+^ OPSCC^41^.

Overall, our findings suggest that HPV mRNA-LNP vaccination in combination with immune checkpoint blockade can sustain the expansion and effector function of HPV-specific CD8^+^ T cells, thereby promoting tumor regression. This combination therapy may be a promising approach for immunotherapy against HPV^+^ OPSCC.

## Methods

### Mice

Wild-type male C57Bl/6J mice were provided by Byrness Weil biotech Ltd (Chongqing, China) and housed in pathogen-free conditions with unlimited access to water and food. The mice used in this study were 6 and 8 weeks old. All animal experiments were approved by the Animal Ethics Committee of the West China Hospital (approval number:20220511001). The experiments complied with the ethical guidelines of the Guide for the Care and Use of Laboratory Animals set by the China Association of Laboratory/Animal Care. And at the defined endpoints, blood was collected from retro-orbital venous plexus under an isoflurane delivery system, animals were then humanely euthanized via cervical dislocation, and spleens, tumor and lymph nodes were harvested for further immunological analysis.

### Formulation of mRNA-loaded LNPs

The HPV-16 E7 protein was designed on the basis of a codon-optimized sequence supplied by WestVac BioPharma Co., Ltd. To synthesize the LNPs, the ethanol phase containing lipids and cholesterol was mixed with the aqueous phase containing mRNA under a microfluidic device. Briefly, 13.5 mM citric acid buffer (pH3) and mRNA were used to prepare the aqueous phase. And DLin-MC3-DMA, DSPC, cholesterol, and DMG-PEG-2000 were mixed at molar ratios of 50, 10, 38.5, and 1.5% to prepare the ethanol phase. Then the two distinct phases were mixed in a 3:1 ratio under a microfluidic device, dialyzed using 10 mM citric acid buffer at pH = 6 for 2 h, and then sterilized with a 0.22 μm filter.

### mRNA-LNP characterization

HPV mRNA-LNPs were characterized using a Zetasizer Nano ZS90 (Malvern Instruments, Malvern, United Kingdom), measuring the average particle size, zeta potential and PDI. The data were obtained as the average of three cates. Meanwhile, a transmission electron microscope (HT7800, Hitachi, Japan) was used to identify the morphology of the mRNA-LNPs.

### In vivo biodistribution of Luc mRNA-LNPs

In vivo imaging system (Caliper Life Sciences) was used to track the time-course biodistribution of luciferase activity according to the manufacturer’s protocols. Following the vaccination with Luc mRNA-LNPs, the mice were photographed at 6 h, 24 h, 48 h, 72 h and 120 h, and a luciferase imaging system was used to determine the luciferase activity. Before imaging, mice were intraperitoneally injected with d-luciferin potassium salt (MeiLunBio) at a dose of 150 mg/kg. Besides, mice were euthanized 6 h following the vaccination with Luc mRNA-LNPs, and a luciferase imaging system was used to determine the luciferase activity in the heart, liver, spleen, lung, and kidney. Living Image® 4.3.1 Software (https://www.perkinelmer.com) was used to measure the average radiance of the region of interest (ROI).

### Cell line

The mEERL cell line is commonly used for HPV^+^ syngeneic mouse models^42,43^. Initially isolated from the oropharyngeal epithelium of a C57BL/6J mouse, it is commonly used in HPV-positive syngeneic mouse models (Richmond, Canada). The mEERL cells were grown in Prigrow IV medium (Cat. No. TM004.) comprised of 10% fetal bovine serum, 0.5 µg/ml hydrocortisone, 5 µg/ml transferrin, 1.36 ng/ml tri-iodo-thyonine, 5 µg/ml insulin, 5 ng/ml epidermal growth factor and 1% penicillin/streptomycin solution. Stocks of mEERL were generated upon receipt of the cells and used for the tumor experiments. Cells were tested regularly for mycoplasma contamination, and none tested positive throughout the study.

### Tumor implantation

For tumor implantation, a frozen cell aliquot was thawed and cultured in the suitable medium at 37 °C and 5% CO_2_, passaged once, and collected using trypsin EDTA (Gibco). Then 2 × 10^6^ cells in 100 μl sterile PBS per mouse were implanted subcutaneously on the right flank. The tumors were measured every 2–3 days using digital calipers. Tumor volume was estimated using the following formula: (tumor volume = Π/6 × length × width^2^). Animals were sacrificed when tumors surpassed 1000 mm^3^ or ulceration was noted.

### Immunization and immune check point blockade

C57Bl/6J non-TB mice were administered with HPV mRNA-LNP (10 μg/100 μl) subcutaneously or intravenously at day 0, 5, and 10 (day 0 as experiment start point). TB mice were allowed to grow for 5–7 days after tumor challenge (day 0), randomized by tumor volume (50–100 mm^3^), and assigned to the treatment or control groups (sample size based on historical data). Mice were injected intravenously with 100 μl PBS, vaccine, and LNP vector at day 9, 14, and 19 post tumor implantations, respectively. For antibody treatment, mice were treated intraperitoneally with anti–LAG3 (murine IgG1, clone C9B7W, BioXcell, 200 μg per dose) or anti–CTLA-4 (murine IgG2, clone 9H10, BioXcell, 100 μg per dose) starting on day 5 with 5 additional times at 3-day intervals.

### Biochemical analysis

Blood was sampled from the retro-orbital plexus of TB mice on day 25. Serum levels ALT, AST, BUN, and CREA were immediately measured using a Roche Cobas c702 analyzer. Data were analyzed using GraphPad Software v8.4.2.

### Cell sorting for scRNA-seq and library construction

Tumors from TB mice mock-vaccinated with PBS and their counterparts vaccinated with HPV mRNA-LNPs were collected on day 25 (6 days after the third dose of vaccination). Spleens from non-TB mice mock-vaccinated with PBS and their counterparts vaccinated with HPV mRNA-LNPs were also collected on day 16 (6 days after the third dose of vaccination) as a control. Spleens were mechanically smashed and washed through a 70 μm cell strainer with culture medium. After centrifugation at 500 × *g* for 5 min, red blood cells in the spleen were lysed using red blood cell lysis buffer (Solarbio). The tumors were cut into small pieces, 2–4 mm^3^. Samples were then dissociated into single-cell suspensions using a Tumor Dissociation Kit (Miltenyi Biotec), according to the manufacturer’s recommendations. Cells were assessed for viability with Zombie NIR™ LIVE/DEAD Fixable Viability Stain kit (BioLegend) for 30 min at 4 °C. The samples were washed and blocked with anti-CD16/CD32 (BioLegend). Cells were simultaneously stained with anti-CD3 (clone 17A2) purchased from BioLegend. After a 30-min incubation at 4 °C, cells were washed twice and resuspended in PBS. CD3^+^ T-cells from the spleen and tumors were sorted using a BD FACSAria SORP Flow Cytometer. All the sorted cells were loaded onto a chromium single-cell sorting system (10× Genomics).

Single-cell transcriptional and VDJ library construction was performed using the Chromium Next GEM Single Cell 5’ Reagent Kit v2, according to the manufacturer’s protocols. The completed libraries were sequenced on a NovaSeq 6000 platform (Illumina).

### Data processing for scRNA-seq

Upstream analysis of scRNA-seq data was performed using *cellranger count* function of the Cell Ranger software (10× Genomics, V3.1.0) with default parameters to align sequencing reads in FASTQ files to the mm10 mouse reference transcriptome and generate a gene-cell matrix, which was input into the Seurat R package (V4.0)^44^ for further analysis and visualization. Based on the standard Seurat pipeline, cells with more than 2500 genes or less than 200 genes and over 5% mitochondrial genes were filtered to obtain high-quality cells for subsequent processing.

### Downstream analysis of scRNA-seq data

*NormalizeData*, *ScaleData*, and *FindVariableFeatures* functions of Seurat with default parameters were applied prior to dimensional reduction using *RunPCA*; all mouse sample datasets were merged together after following the same procedures. Considering the batch effect between mouse samples, the function *RunHarmony* embedded in the harmony R package (V0.1.0)^45^ was used to reveal clearer biological differences. Combined data were normalized, scaled, and principal components were computed. The top 30 principal components were selected for unsupervised clustering with *FindNeighbors* and *FindClusters* function (resolution = 0.5). Non-linear dimensional reduction was visualized using t-distributed Stochastic Neighbor Embedding (t-SNE). All clusters were annotated based on canonical gene markers; CD8^+^ T cell-specific clusters (Cd3e^+^ Cd8a^+^) were extracted for further sub-clustering analysis. The extracted 19,938 CD8^+^ T cells were obtained from the normalized transcriptome data, which produced 16 clusters (resolution = 1.0). The differentially expressed genes of each cluster were explored using the *FindAllMarkers* function with min.pct set to 0.25 and logfc.threshold set to 0.25 (Supplementary Data 1); those with avg_log2FC > 0.5 and p_val_adj < 0.05 were considered cluster-specific genes. Subsequently, the top 20 marker genes combined with the classic genes were used to annotate the CD8^+^ T cell subtype.

### Supervised trajectory inference

To explore the trajectory of CD8^+^ T cells, SingleCellExperiment (V1.16.0)^46^ was used to reconstruct the cell-gene expression matrix from a combined dataset embedded in t-SNE reduction and subset the assigned cell group by sub-clusters for downstream analysis. Then *slingshot* and *as.SlingshotDataSet* functions of the slingshot (V2.2.1)^47^ R package were used to identify the global lineage structure, fit simultaneous principal curves to describe each lineage, and visualize the trajectories.

### TCR-sequencing analysis

Single-cell TCR sequencing raw data produced from Chromium Single Cell 5’ V(D)J libraries were analyzed using the *cellranger vdj* pipeline of Cell Ranger (V3.1.0) with default parameters to align sequencing reads in FASTQ files to the mm10 mouse reference transcriptome and generate complementarity determining region (CDRs) annotation files containing filtered_contig_annotations, which were used as the input for further analysis. The scRepertoire (V1.3.5)^48^ R package was used to assign a clonotype based on two TCR or Ig chains and analyze T cell clonotype dynamics.

### Tissue processing and single-cell suspension preparation for flowcytometry

For CD8^+^ T-cell flow cytometry analysis in non-TB mice, the spleen, blood, and inguinal lymph nodes were collected. For CD8^+^ T-cell analysis in TB mice, the spleen, blood, draining lymph nodes, and tumors were collected. Heparin-treated blood samples were collected and lysed using red blood cell lysis buffer (Solarbio). Spleens and lymph nodes were mechanically smashed and washed through a 70 μm cell strainer with culture medium. After centrifugation at 500 × *g* for 5 min, red blood cells in the spleen were lysed using red blood cell lysis buffer (Solarbio). The tumors were cut into small pieces, 2–4 mm^3^. Samples were dissociated into single-cell suspensions using a Tumor Dissociation Kit (Miltenyi Biotec), according to the manufacturer’s recommendations. Cell pellets were resuspended in PBS for subsequent experiments.

### Flowcytometry

Cells were assessed for viability with Zombie NIR™ LIVE/DEAD Fixable Viability Stain kit (BioLegend) for 30 min at 4 °C. The samples were washed and blocked with anti-CD16/CD32 (BioLegend). For CD8^+^ T cells analysis in non-TB mice, cells were simultaneously stained with the following surface antibodies: CD8a (clone 53-6.7) BV510 diluted 1:40 (BioLegend, cat. 100752), CD44 (clone IM7) PerCP/Cyanine5.5 diluted 1:80 (BioLegend, cat. 103032), CD62L (clone MEL-14) AF700 diluted 1:200 (BioLegend, cat. 104426), PD-1 (clone RMP1-30) PE/Cyanine7 diluted 1:20 (BioLegend, cat. 109110), KLRG1 (clone 2F1/KLRG1) BV711 diluted 1:80 (BioLegend, cat. 138427), IL-7Rα (clone A7R34) BV605 diluted 1:20 (BioLegend, cat. 135025), Ly108 (clone 330-AJ) APC diluted 1:40 (BioLegend, cat. 134610), and MHC I Dextramer (RAHYNVTF/H-2 Db) PE (IMMUDEX, cat. JA2195) (1:20 dilution) specific for the E7 antigen. For CD8^+^ T cell analysis in TB mice, the cells were simultaneously stained with the following surface antibodies: CD45 (clone 30-F11) BV711 diluted 1:80 (BioLegend, cat. 103147), CD3 (clone 17A2) BV421 diluted 1:20 (BioLegend, cat. 100227), CD8a (clone 53-6.7) BV510 diluted 1:40 (BioLegend, cat. 100752), PD-1 (clone RMP1-30) PE/Cyanine7 diluted 1:20 (BioLegend, cat. 109110), IL-7Rα (clone A7R34) BV605 diluted 1:20 (BioLegend, cat. 135025), and MHC I Dextramer (RAHYNVTF/H-2 Db) PE (IMMUDEX, cat. JA2195) (1:20 dilution) specific for the E7 antigen.

After a 30-min incubation at 4 °C, cells were washed twice in cell staining buffer, fixed, and permeabilized using the Transcription Factor Buffer Set (BD Biosciences). The fixed cells were washed and intracellularly stained with Ki-67 (clone 11F6) AF488 diluted 1:200 (BioLegend, cat. 151204) for 50 min in the dark at 4 °C. After washing twice, the cells were resuspended in PBS and measured using a BD FACSAria SORP Flow Cytometer.

### Dimension reduction and clustering of multiparameter flowcytometry

For examining phenotypes of CD8^+^ T cell subsets in non-TB mice, flowcytometry data of CD8^+^ T cells in spleen, blood, and inguinal lymph nodes after different treatments were concatenated, and processed for FltSNE plugins (max iterations = 1000, perplexity = 20 and learning rate = 200, 10.48550/arXiv.1712.09005) using the parameters of CD8a BV510, CD44 PerCP/Cyanine5.5, CD62L AF700, PD-1 PE/Cyanine7, KLRG1 BV711, IL-7Rα BV605, Ly108 APC, Ki-67 AF488, and MHC Dextramer PE in FlowJo v.10.8.1 (BD Biosciences). For examining phenotypes of CD8^+^ T cell subsets in TB mice, flowcytometry data of CD8^+^ T cells of spleen, blood, tumor, and draining lymph nodes after different treatments were concatenated, and processed for FltSNE plugins using the parameters of CD45 BV711, CD3 BV421, CD8a BV510, PD-1 PE/Cyanine7, IL-7Rα BV605, Ki-67 AF488, and MHC Dextramer PE in FlowJo v.10.8.1 (BD Biosciences).

### Statistical analysis

GraphPad Prism 8.4.2 software was used to prepare all graphs and perform statistical analysis, including two-way ANOVA with Tukey’s multiple comparisons test. Error bars indicate standard error means (SEM). *P* < 0.05 was considered as statistically significant, and all statistically significant values in figures are indicated as: *P < 0.05, **P < 0.01, ***P < 0.001, and ****P < 0.0001.

### Supplementary information

Reporting-summary

Supplementary Information

Supplementary Data 1

Supplementary Data 2

Supplementary Data 3

Supplementary Data 4

## Data Availability

Sequence data that support the findings of this study is available via NCBI Sequence Read Archive (SRA) under accession PRJNA914791. The code and scripts are available from the corresponding author on reasonable request.
