# Supplementary material for: mRNA-LNP vaccination-based immunotherapy augments CD8+ T cell responses against HPV-positive oropharyngeal cancer
Source: NPJ Vaccines. 2023 Sep 29;8:144. doi: 10.1038/s41541-023-00733-8 (PMC10542330; doi:10.1038/s41541-023-00733-8)
Supplement: Supplementary file 2 — Supplementary Information [file 41541_2023_733_MOESM2_ESM.pdf]

This Supplementary Information file includes:

Supplementary Figure. 1 to 6

Caption for Supplementary Data.

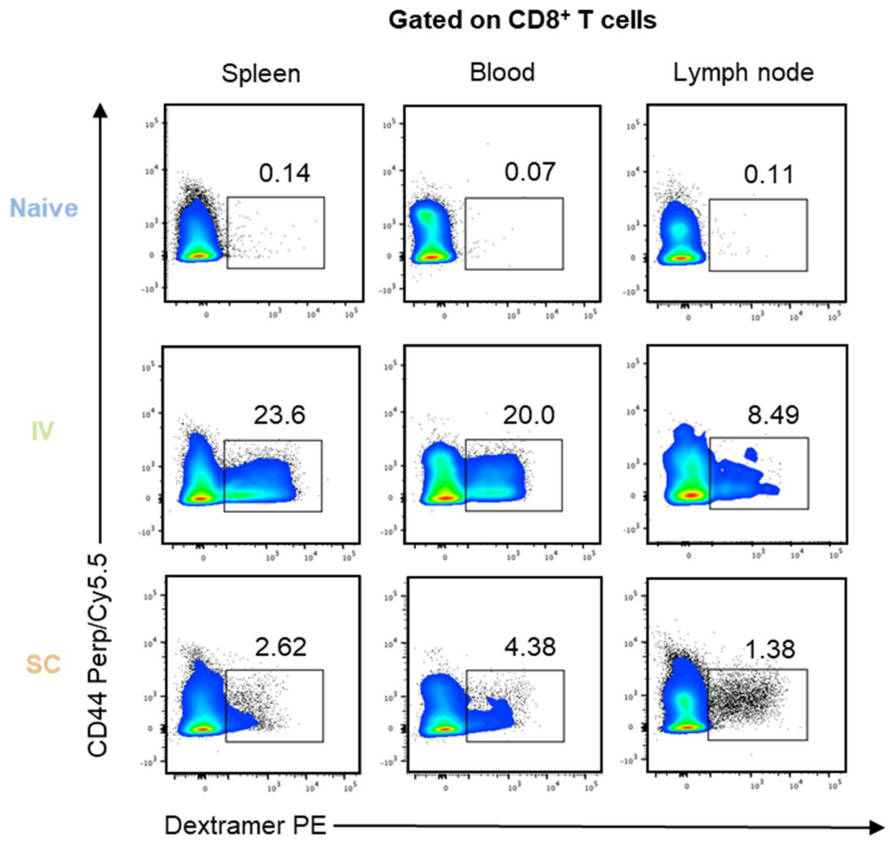

Supplementary Fig. 1: The route of vaccination alters the magnitude of HPV-specific CD8^{+} T cells. (a) Flow cytometric analysis of single cells stained with E7 dextramer and CD44 antibody. The numbers indicate the percentage of cell population within the gate. Naive: unvaccinated; IV: intravenous; SC: subcutaneous.

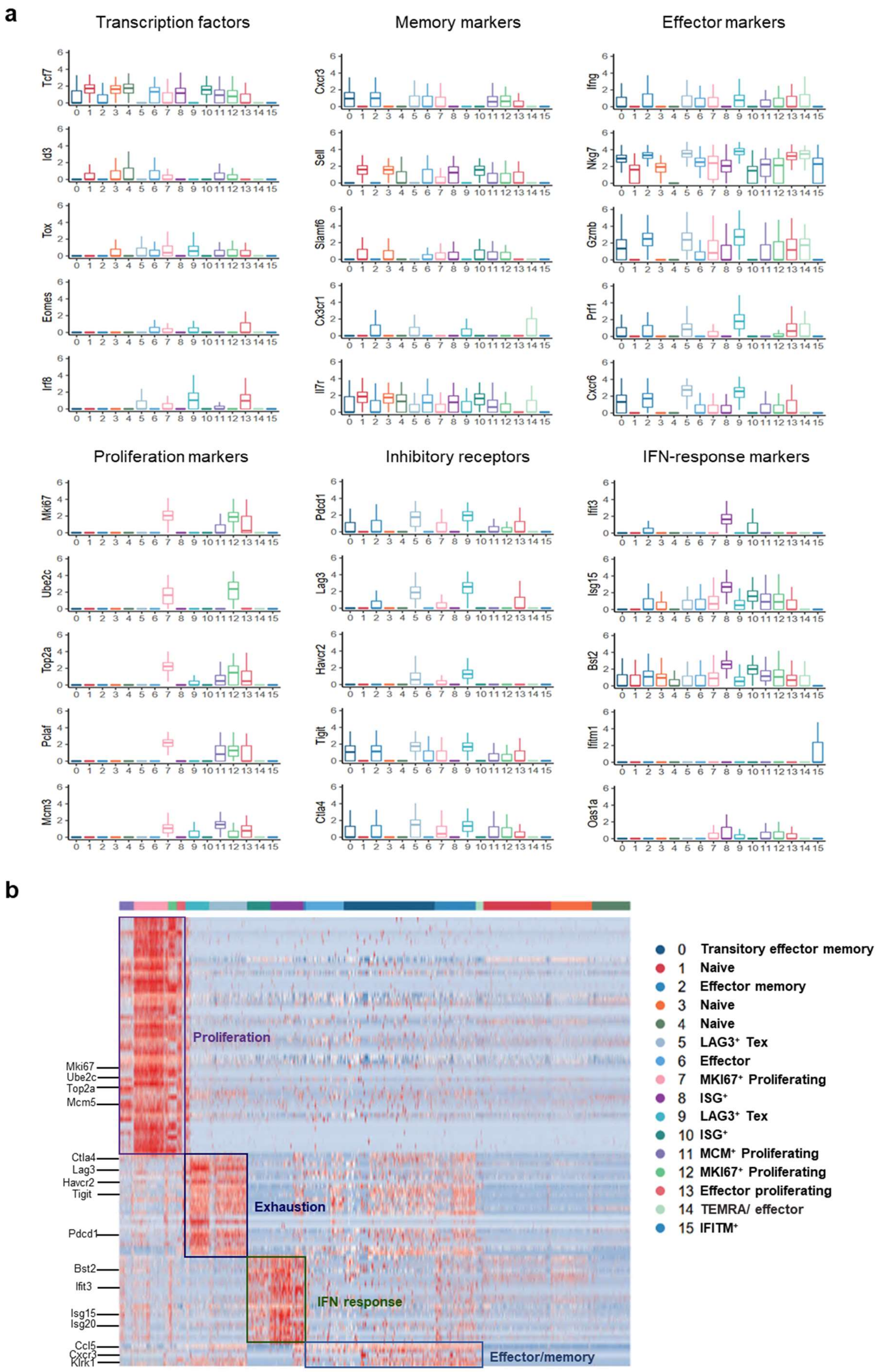
a
Transcription factors
Memory markers
Effector markers
Tcf7
Id3
Tox
Eomes
Irf8
Cxcr3
Sell
Slamf6
Cx3cr1
Il7r
Ifng
Nkg7
Gzmb
Prf1
Cxcr6
Proliferation markers
Inhibitory receptors
IFN-response markers
Mki67
Ube2c
Top2a
Pclaf
Mcm3
Pdcd1
Lag3
Havcr2
Tigit
Ctla4
Ifit3
Isg15
Bst2
Ifitm1
Oas1a
b
Proliferation
Exhaustion
IFN response
Effector/memory
Mki67
Ube2c
Top2a
Mcm5
Ctla4
Lag3
Havcr2
Tigit
Pdcd1
Bst2
Ifit3
Isg15
Isg20
Ccl5
Cxcr3
Klrk1
0 Transitory effector memory
1 Naive
2 Effector memory
3 Naive
4 Naive
5 LAG3+ Tex
6 Effector
7 MKI67+ Proliferating
8 ISG+
9 LAG3+ Tex
10 ISG+
11 MCM+ Proliferating
12 MKI67+ Proliferating
13 Effector proliferating
14 TEMRA/ effector
15 IFITM+

Supplementary Fig. 2: Expression patterns of functional marker genes in different subclusters. (a) The expression of genes encoding transcription factors, inhibitory receptors, memory, effector, proliferation and IFN-response markers across subclusters. The median is shown by the center line within the box of box plots, while the lower and upper box boundaries reflect the 25th and 75th percentiles, respectively, with whiskers drawn to the number that is closest to but still falls within the 1.5 interquartile range from top to bottom of the box borders. **(b)** Heatmap of selected top differentially expressed (DE) genes in each cluster.

a

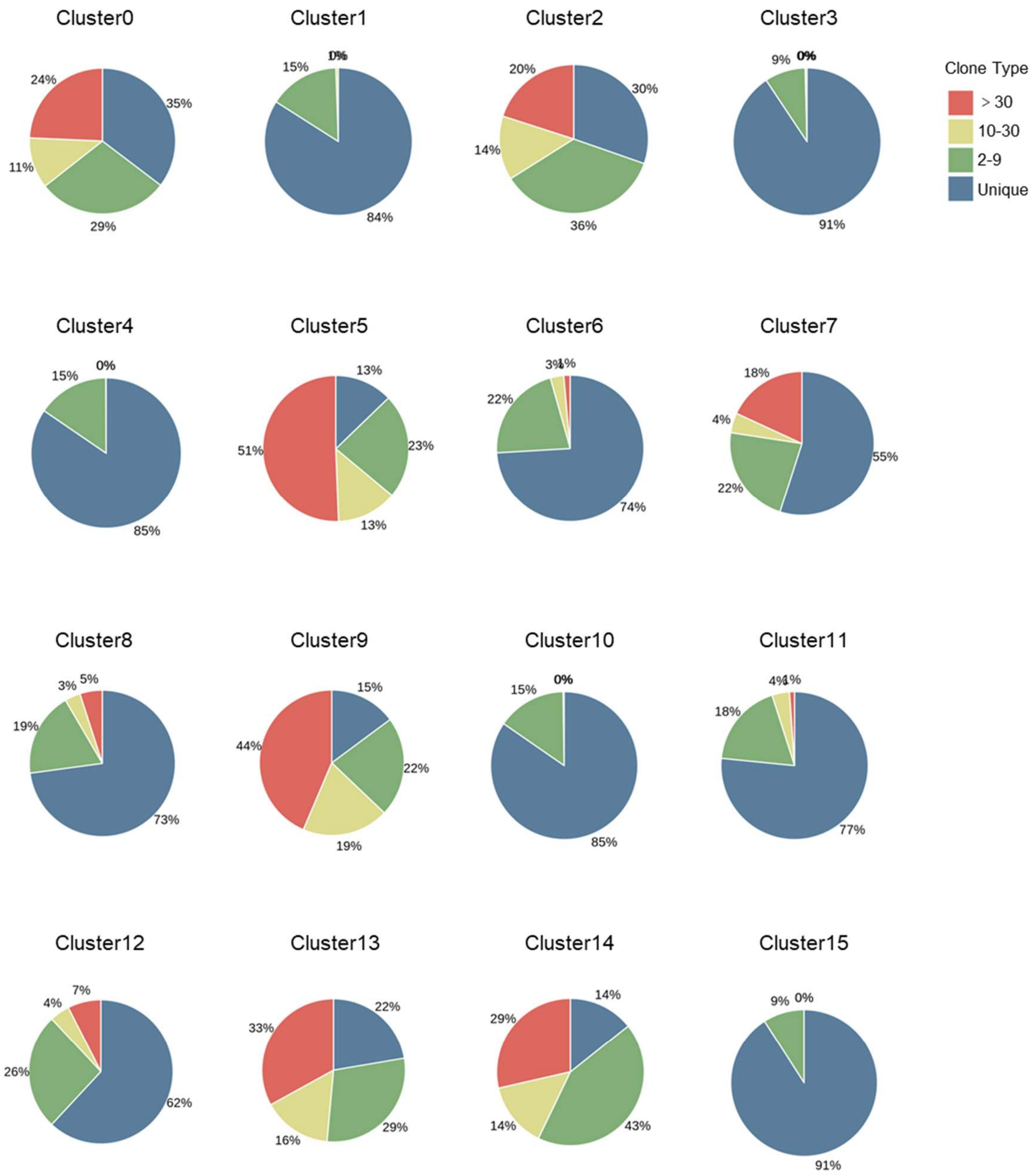

Cluster0
24%
35%
11%
29%
Cluster1
15%
84%
Cluster2
20%
30%
14%
36%
Cluster3
9%
91%
Clone Type
> 30
10-30
2-9
Unique
Cluster4
15%
0%
85%
Cluster5
13%
51%
23%
13%
Cluster6
22%
74%
Cluster7
18%
4%
55%
22%
Cluster8
3%
5%
19%
73%
Cluster9
15%
44%
22%
19%
Cluster10
15%
0%
85%
Cluster11
18%
77%
Cluster12
7%
4%
26%
62%
Cluster13
22%
33%
16%
29%
Cluster14
14%
29%
14%
43%
Cluster15
9%
0%
91%

b

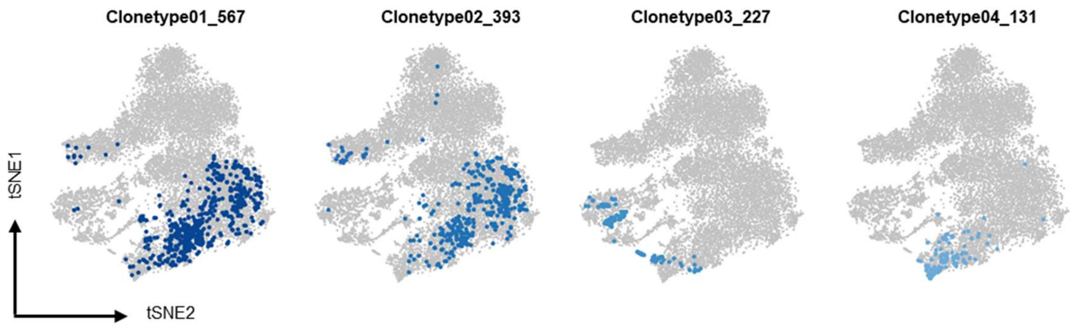

Clonetype01_567
Clonetype02_393
Clonetype03_227
Clonetype04_131
tSNE1
tSNE2

Supplementary Fig. 3: Distribution of TCR clonotypes in subclusters. (a) Pie charts of TCR clonotype size for each cluster. **(b)** Distribution of TOP4 clonotypes projected onto t-SNE maps.

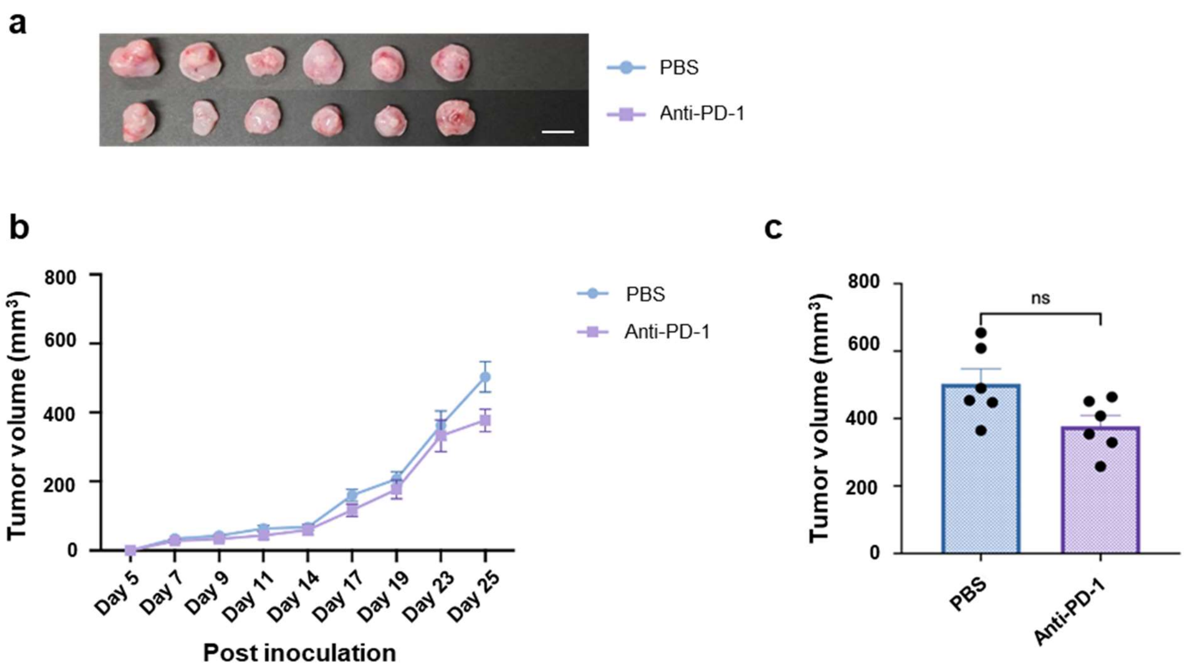

Supplementary Fig. 4: Application of PD-1 blockade in HPV+ OPSCC mouse model. (a) Representative images of tumors collected on day 25 following different treatment. The scale bar indicates 1 cm. **(b-c)** Tumor growth following treatment (n = 6). Statistics were assessed by the Mann–Whitney U test. *P < 0.05, **P < 0.01 and ***P < 0.001. Anti-PD1 (rat IgG2a, k, clone 29F.1A12, BioXcell) was administered intraperitoneally 200 μg per treatment starting on day 5 with 5 additional times at 3-day intervals. Error bar = mean ± SEM.

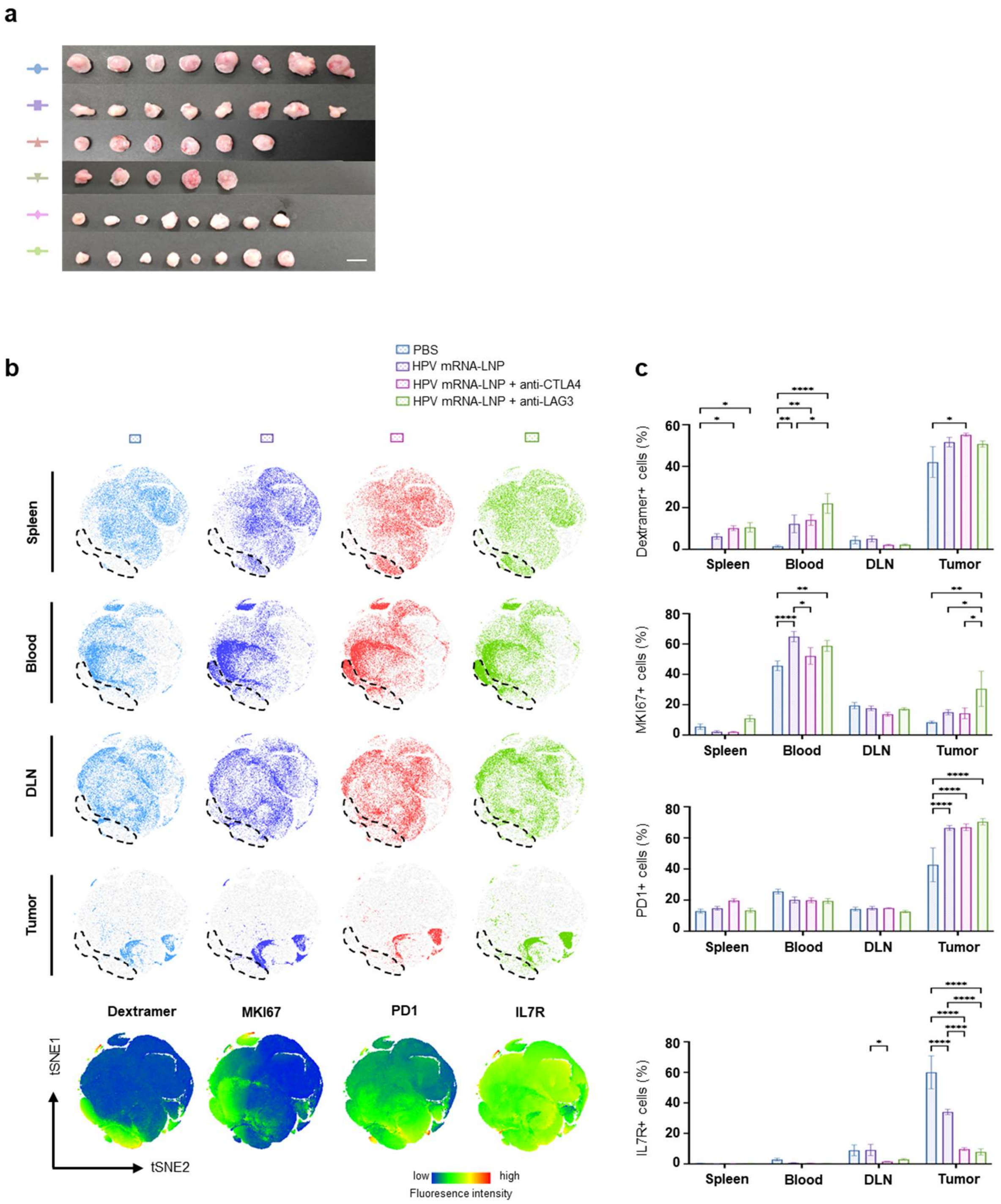

Supplementary Fig. 5: HPV mRNA-LNP vaccination combined with immune checkpoint blockade generates HPV-specific CD8^{+} T cells with superior antitumor capacity. (a) Representative images of tumors collected on day 25 following different treatment. The scale bar

indicates 1 cm. **(b)** Distribution of $CD8^+$ T cells projected onto t-SNE maps using flowcytometry data and t-SNE heatmaps, for each marker applied on $CD8^+$ T cell events. Black circle indicates $dextramer^+$ population **(c)** Expression of different immune markers on $CD8^+$ T cells measured in spleen, blood, DLNs, and tumor (n = 4 ～ 8). DLNs： draining lymph nodes. Statistics were assessed by two-way ANOVA with Tukey's multiple comparison tests. *P < 0.05, **P < 0.01 and ***P < 0.001. Error bar = mean ± SEM.

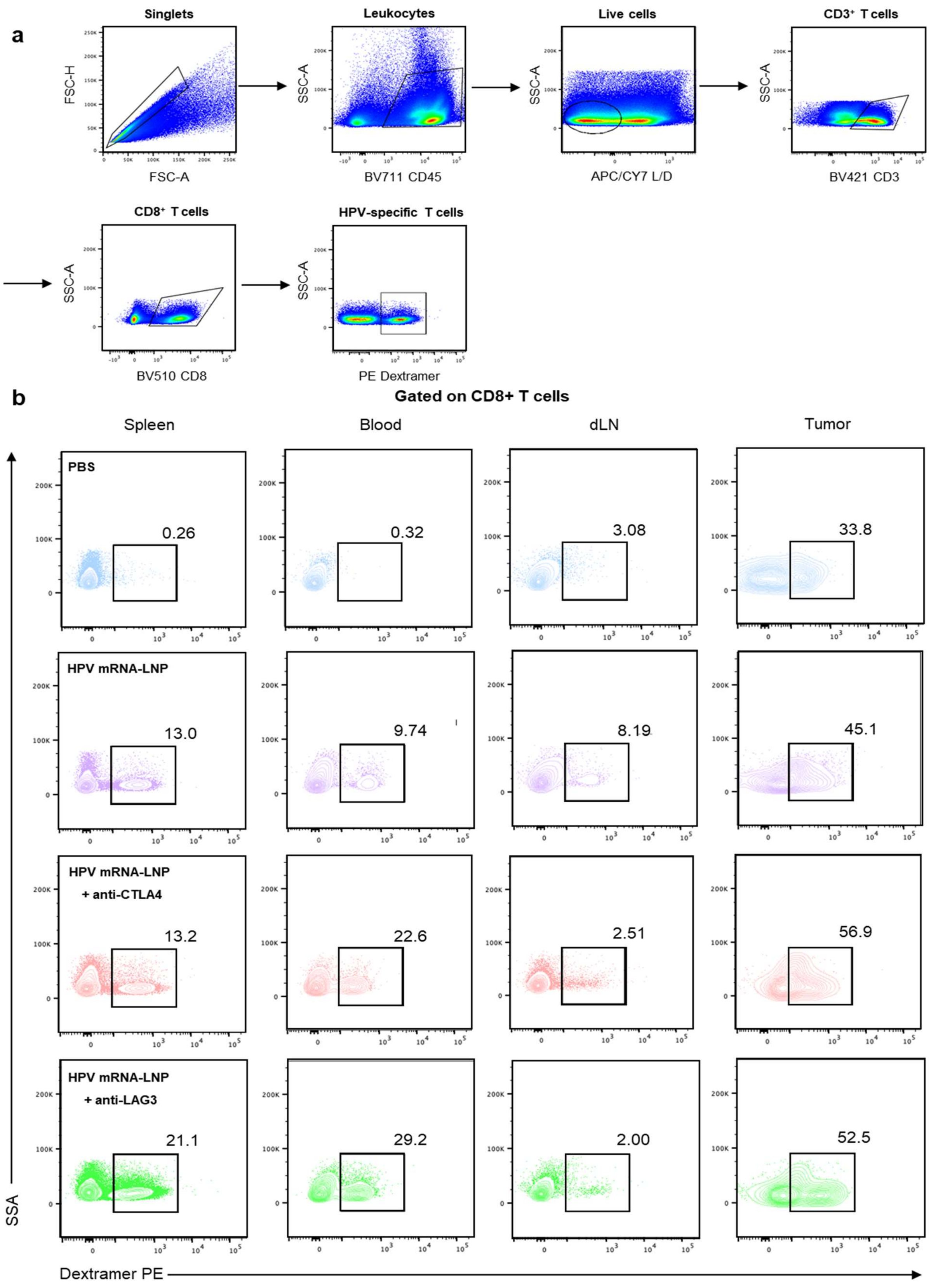
a
Singlets
Leukocytes
Live cells
CD3⁺ T cells
CD8⁺ T cells
HPV-specific T cells
FSC-H
FSC-A
SSC-A
BV711 CD45
APC/CY7 L/D
BV421 CD3
BV510 CD8
PE Dextramer
b
Gated on CD8+ T cells
Spleen
Blood
dLN
Tumor
PBS
0.26
0.32
3.08
33.8
HPV mRNA-LNP
13.0
9.74
8.19
45.1
HPV mRNA-LNP
+ anti-CTLA4
13.2
22.6
2.51
56.9
HPV mRNA-LNP
+ anti-LAG3
21.1
29.2
2.00
52.5
SSA
Dextramer PE

Supplementary Fig. 6: HPV mRNA-LNP vaccination combined with immune checkpoint blockade boosts HPV-specific $CD8^+$ T cells. (a) Gating strategy for sorting HPV-specific $CD8^+$ T cells. **(b)** Flow cytometric analysis of single cells stained with E7 dextramer antibody. The numbers indicate the percentage of $Dextramer^+$ population within the gate.

Supplementary Data 1. Differentially expressed genes of different cell clusters applied in scRNA-seq.

Supplementary Data 2. Cell percentages across treatments and tissues for each trajectory.

Supplementary Data 3. Frequencies and sequences of top expanded clonotypes (clone size ⩾ 30).

Supplementary Data 4.TCR-wise cell counts of top clonotypes (clone size ⩾ 30) across clusters in the spleens and tumors, respectively.
